# A Preliminary Exploration of Transcriptome and Proteomic Changes During the Young and Harvest Periods in *Morchella sextelata*

**DOI:** 10.3390/jof11030192

**Published:** 2025-03-02

**Authors:** Weilin Feng, Zier Guo, Qunli Jin, Fei Xu, Yingyue Shen, Tingting Song, Mei Wang, Jun Zhang, Lijun Fan, Xianbin Huang, Weiming Cai

**Affiliations:** 1 Institute of Horticulture, Zhejiang Academy of Agricultural Sciences, Hangzhou 310021, China; fengwl@zaas.ac.cn (W.F.); guoze@zaas.ac.cn (Z.G.); jinql@zaas.ac.cn (Q.J.); shenyingyue@zaas.ac.cn (Y.S.); songtingting@zaas.ac.cn (T.S.); wangmei@zaas.ac.cn (M.W.); zhangjun@zaas.ac.cn (J.Z.); fanlj@zaas.ac.cn (L.F.); h2674395715@outlook.com (X.H.); 2 College of Biology and Environment, Zhejiang Wanli University, Ningbo 315100, China; 3 State Key Laboratory for Managing Biotic and Chemical Threas to the Quality and Safety of Agro-Products, Zhejiang Academy of Agricultural Sciences, Hangzhou 310021, China; fxu@zaas.ac.cn; 4 College of Advanced and Agricultural, Zhejiang A&F University, Hangzhou 311300, China

**Keywords:** RNA-seq, selenium, *Morchella sextelata*, glycometabolism

## Abstract

Based on transcriptome and proteome sequencing technologies, this study aims to preliminarily reveal the molecular mechanisms of growth and development and related metabolic regulation in *Morchella sextelat*. A total of 42.31 GB of Clean Data was acquired from the transcriptome sequencing (RNA-seq) of six samples in two development phases (*n* = 3) of *M. sextelata.* In the young phase (YP) and harvest phase (HP), there were 2887 differentially expressed genes (DEGs), including 1910 up-regulated genes and 977 down-regulated genes. In YP and HP, there were 987 differentially expressed proteins (DEPs), including 417 up-regulated ones and 570 down-regulated ones. Based on GO and KEGG analysis, significant differences in the transcriptomes and proteins in metabolic pathways are disclosed. Glycometabolism, especially starch, saccharose, and polysaccharide metabolism, plays a crucial role in the growth of *M. sextelata.* In addition, expression changes in the genes related to selenium metabolism are here recognized. These research results not only offer strong support for further exploration of the biological significance and functional differences of *M. sextelata*, but are also conducive to discovering key genes and understanding their regulation network during growth.

## 1. Introduction

*Morchella sextelata* belongs to *Morchellaceae*, *Pezizales*, *Pezizomycetes*, and *Ascomycota* [1]. *M. sextelata* is a black morel mushroom variety that has been successfully domesticated under the field cultivation mode in China in recent years, and the species’ name relates to the number *Mel-6* in its phylogenetic classification. It is a type of medical and edible fungi that is extensively distributed across the world. Some modern medical studies have proven that *M. sextelata* is rich in multiple bioactive substances related to tumor resistance [2,3], aging resistance [4,5,6], bacterial resistance [7,8], liver protection [9], lipid-lowering, and immunity enhancement. Nevertheless, the spontaneous generation of *M. sextelata* is notably seasonal, and its wild resources are decreasing dramatically due to overpicking. Although significant progress has recently been achieved in field production technologies for *M. sextelata*, acquiring a stable and high yield still faces many technological challenges. In particular, there are few basic studies on the growth, development, yield, and quality formation of *M. sextelata* [10]. Through transcriptomics analysis, researchers have recognized the relevant genes of *M. sextelata* related to drought stress; the results identified 18 key differentially expressed genes (DEGs) associated with drought stress, including transporter proteins, thermokines, transcription factors, kinases, and autophagy-related genes [11]. In studies of other edible mushrooms, transcriptomics has been applied to explore gene expression modes in different development phases. For example, the transcriptomic analysis of the fruiting bodies of *Agaricus bisporus* in different development stages disclosed expression changes in genes related to development and reproductive processes [12,13]. Similarly, proteomics analysis provides protein information after gene expression by analyzing the expression and modification of intracellular proteins. This is crucial to understanding gene functions and metabolic pathways. For instance, transcriptomic and proteomic analyses on the stipe development of *Flammulina velutipes* have provided important information that will help us understand the molecular mechanisms at play in the development process [14].

In studies of *M. sextelata*, the young phase (YP) and harvest phase (HP) have been determined as critical stages in its life cycle. Changes in gene expression and protein synthesis in these two phases are not only directly related to the shape formation and nutrient accumulation of *M. sextelata*, but also have direct impacts on the shape formation, sugar accumulation, and production of antioxidant substances in *M. sextelata.* Recently, research methods using transcriptomics and proteomics have provided strong tools to analyze the growth and development mechanisms of *M. sextelata.* For example, through transcriptome analysis, researchers disclosed key functional metabolic pathways involving ABC transporters, amino acid metabolism, and the biosynthesis of fatty acids and metabolic pathways important during the growth and development of *M. sextelata* [15]. The multi-omics joint analysis approach proposed by Chen et al. (2023) [16] is also employed to achieve a deep understanding of the shape formation and maturation process of *M. sextelata.* It emphasizes the significant correlation between differentially expressed miRNA (DEM) and differentially expressed genes (DEGs). Moreover, specific amino acid and nucleotide metabolic pathways may play a critical role in the shape development and maturation of the ascocarp. Following recent studies on genomics, the PacBio genomic sequencing of *M. sextelata* has provided us with deeper genomic information [17,18]. In addition, the effects of environmental factors like temperature on the antioxidase activity and gene expression of *M. sextelata* have also attracted some attention [19]. It was discovered, through a deep sequencing analysis of the genomes of *Morchella importuna*, that different mating types may differ in their genomes, genes, and functional layers. Combined with cultivation experiments on monospore and hybrid groups, this may verify that *M. importuna* is a type of heterothallic fungus. Moreover, a transcriptome sequencing (RNA-seq) analysis of the nutrient growth stage from the hypha to the sclerotium has been completed [20,21]. Hao et al. (2019) [22] carried out transcriptome de novo sequencing in the hypha phase and young phase of *M. importuna* and analyzed the DEGs in these two phases. Chen et al. (2024) [23] discussed the nutrient quality in *M. sextelata* cultivated on a plateau and found that vitamins B6, B9, D, and E had extremely significantly negative correlations with the total essential amino acids, while B6 showed a significantly positive correlation with the crude polysaccharide content. This proves that vitamins might influence nitrogen metabolism and glycometabolism in *M. sextelata* directly under environmental stress.

In this study, genes in *M. sextelata* related to carbohydrate metabolism, glycometabolism, and antioxidation were further explored through transcriptome–proteome joint analysis, aiming to provide new theoretical references and practice guidance for the cultivation, quality improvement, and functional product development of *M. sextelata.* Deep studies on the functions and regulation mechanisms of these genes are expected to open a new approach to the sustainable development and extensive application of *M. sextelata.*

## 2. Materials and Methods

### 2.1. Test Strains

The *M. sextelata* provided by Sichuan Junzhiwei Agricultural Development Co., Ltd. (Chengdu, China) was chosen as the test strain. The field cultivation and management of samples were carried out at the Yangdu Base of Zhejiang Academy of Agricultural Sciences using conventional field cultivation technology for *M. sextelata*, named “Yang30”, at the base. To develop from nutritive growth to reproductive growth, the mycelium of *M. sextelata* requires the stimulation of temperature and humidity differences. Morel mushrooms are cultivated in loamy soil with mycelial growth temperatures of 10~20 °C and mushrooming temperatures of 5~16 °C. The *M. sextelata* was manually divided into seven phases, including the primary base phase, bud phase, young phase, small-size phase, harvest phase, maturity phase, and post-maturity phase. To increase the significant differences, two phases were chosen for sampling during the period of fruit body development, namely, YP (obvious differentiation between pileus and stipe, ascocarp height—4.5–5.5 cm) and HP (obvious differentiation between pileus and stipe, ascocarp height—13.5–14.5 cm). Roots and dirt on the fresh fruiting body samples were removed. Pilei were collected from YP and HP with a sterile scalpel. All samples were then sealed with aluminum foil and then transferred immediately to liquid nitrogen. They were then frozen in a refrigerator at −80 °C for later use. Each sample underwent three repetitions.

### 2.2. Reference Genomic Data

The reference genes were from Morchella_sextelata. The reference genome version was GCF_020137385.1.

The reference genomes were from https://www.ncbi.nlm.nih.gov/datasets/genome/GCF_020137385.1/ (accessed on 2 November 2024).

The transcriptome data were submitted to NCBI (http://www.ncbi.nlm.nih.gov/, accessed on 2 December 2024), with the login number PRJNA1189673 (BioProject). The proteomic data were stored in the ProteomeXchange Consortium via iProX (http://proteomecentral.proteomexchange.org, accessed on 28 November 2024), with the PDX number PXD058217.

### 2.3. Total RNA Extraction and RNA Sequencing from Fruiting Body of M. sextelata

Fruiting body samples of *M. sextelata* were mixed and then ground (*n* = 3). Total RNA was extracted using the total RNA extraction kit (TRIzol^®^ Reagent) according to the manufacturer’s instructions, and the concentration and purity of RNA were tested by the UV-light adsorption method. The integrity of RNA was tested by agarose gel electrophoresis. Magnetic beads were added to the extracted total RNA, and the further enrichment of mRNA was realized through base pairing. Subsequently, a segmented buffer solution was added to break the mRNA into small segments (about 200 bp), which were used as templates. The first chain of cDNA was synthesized by using random primers and reverse transcriptase. Subsequently, the second chain of cDNA was synthesized, deriving the double cDNA of cohesive terminals. Later, the terminal repair reagent was added to form flat terminals. The DynabeadsTMmRNA purification kit (Thermo Fisher Scientific, Waltham, MA, USA), library building kit (Truseq TM RNA Sample Prep Kit), bridge amplification kit (Novaseq Xplus PE Cluster Kit) (Illumina, CA, USA) and library recovery reagent (Certified Low Range Ultra Agarose) (Bio-Rad, Hercules, CA, USA) were used. Moreover, a basic group was added at the 3′ terminal and connected to the sequencing connector. The cDNA library was built and amplified by using the library building kit and bridge amplification kit, respectively. Then, it was loaded onto a computer for sequencing (Novaseq Xplus sequencing system). In this study, cDNA building and sequencing services were provided by Shanghai Majorbio Co., Ltd. (Shanghai, China).

### 2.4. RNA-Seq Analysis

The raw reads derived through RNA-seq contain some repeated and low-quality reads with joints, which are disadvantageous for subsequent comparisons and analyses. Hence, refined filtering is required to acquire high-quality, clean reads. During filtering, read pairs with n > 3, a mass lower than 5, and a proportion ≥ 20%, and reads containing an adapter were eliminated. The raw paired-end reads were trimmed and quality-controlled using fastp [24] with default parameters. Then, the clean reads were separately aligned to the reference genome via orientation mode using the HISAT2 v2.2.1 [25] software. The mapped reads of each sample were assembled by StringTie [26] via a reference-based approach. To study the gene expression changes among samples or groups, a gene expression matrix was built. Subsequently, the levels of significance of the differential expressions of read count data were analyzed by edgeR software v4.0.2, including normalization to eliminate systematic bias, calculating the *p*-value to evaluate the significance of differences, and making multiple hypotheses of the testing calibration to derive a *q*-value. To control the false-positive rate, *q*-value and fold change were combined for differential gene screening, with standards of |log_2_(Fold Change(FC))| > 1 and a *q*-value < 0.05. If the number of differentially expressed genes (DEGs) was lower than 100, the standards were adjusted to |log_2_(FC)| > 1 and *p*-value < 0.05. Essentially, differential expression analyses were performed using the DESeq2 [27].

### 2.5. Proteome Sequencing

RNA-seq was employed, and proteome sequencing was carried out by Shanghai Majorbio Co., Ltd. (Shanghai, China). The main process is as follows: First, we extracted total proteins from the samples. Some total proteins were collected to test protein concentration, and SDS-PAGE was performed for quality control. Next, trypsin enzymolysis was carried out, and equal amounts of samples were collected to produce a chromatograph. Finally, test and bioinformatic data analyses were conducted using an Orbitrap Astral mass spectrometer (Thermo) in Data Independent Acquisition (DIA) mode. *p*-values and FC values for the proteins between the two groups were calculated using R package “*t*-test”. The thresholds of FC (>1.2 or <0.83) and *p*-values < 0.05 were used to identify differentially expressed proteins (DEPs).

### 2.6. Bioinformatics Analysis and Data Analysis

The bioinformatic analysis of proteomic data was performed on the Majorbio Cloud platform (https://cloud.majorbio.com) (accessed on 7 November 2024) [28,29]. The functional annotation of all identified proteins was performed using GO [30] (http://geneontology.org/, accessed on 17 November 2024) and KEGG pathway analysis [31] (http://www.genome.jp/kegg/, accessed on 17 November 2024). DEPs and DEGs were further used for GO and KEGG enrichment analysis. Protein–protein interaction analysis was performed using the String v11.5 (https://string-db.org, accessed on 17 November 2024). Three biological replicates were set for each sample. Three data analysis software applications, SPSS v26, Graphpad Prism v9.5.1, and Excel v2016, were used to process and interpret the experimental data. Primers were designed using the software Primer Premier v5.0. The statistical analysis was performed using an Excel 2016 test. The datasets are presented as means ± standard deviation.

### 2.7. Real-Time Fluorescence Quantitative PCR Verification

To further verify the accuracy of the transcriptome data, DEGs related to cell wall structure, metabolism, and carbohydrate metabolism were tested for expression using the RT-qPCR method. The 18S was used as the internal reference gene. In this qPCR test, the 2×qPCR MIX (Art. No. Q421-02) provided by Nanjing Novizan Company (Nanjing, China) was applied. The reaction system was repaired according to the following components and their volumes: 7.7 μL nuclease-free water, 10 μL 2×qPCR MIX, 0.4 μL preprimer (concentration: 10 μM), 0.4 μL after-primer (concentration: 10 μM), and 1.5 μL DNA template, with a total volume of 20 μL. The amplification program included 5 min of pre-degeneration at 95 °C for one cycle, 15 s of degeneration at 95 °C for 40 cycles, and 30 s of annealing/extension at 55 °C for 40 cycles. Details of primer design for candidate genes are shown in Supplementary Table S1.

## 3. Results

### 3.1. RNA-Seq Data Quality

**Figure 1:**
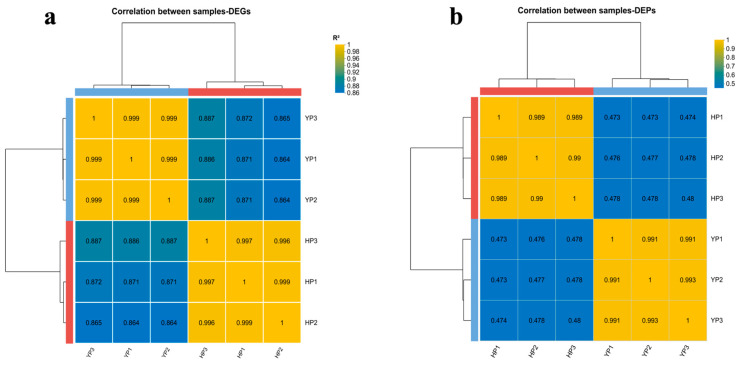
Heat maps of correlation: (**a**) DEGs and (**b**) DEPs.

A total of 42.31 GB of Clean Data was collected from six samples. It can be seen in Supplementary Table S1 that the Q20 was 98.76~98.85%, the Q30 was 96.05~96.32%, and the proportions of guanine and cytosine were 48.92~49.70%. According to the expression values (Transcripts Per Kilobase of exon model per Million mapped reads, TPM) of different sampled genes, correlation and principal component analyses of gene levels were carried out. The correlation coefficient (R^2^) among samples was calculated. If R^2^ was closer to 1, the similarity of the gene expression mode between samples was higher. In Figure 1, the inter-group sample difference and intra-group sample repetition are displayed intuitively. Principal component analysis (PCA) was performed at the gene level based on the expression values (TPM) of the genes in each sample (Supplementary Figure S1). Hence, it can be seen from the following table that the sample and the overall sequencing quality were good, and the data can be used for follow-up bioinformatics analysis.

### 3.2. Analysis of DEGs and DEPs

**Figure 2:**
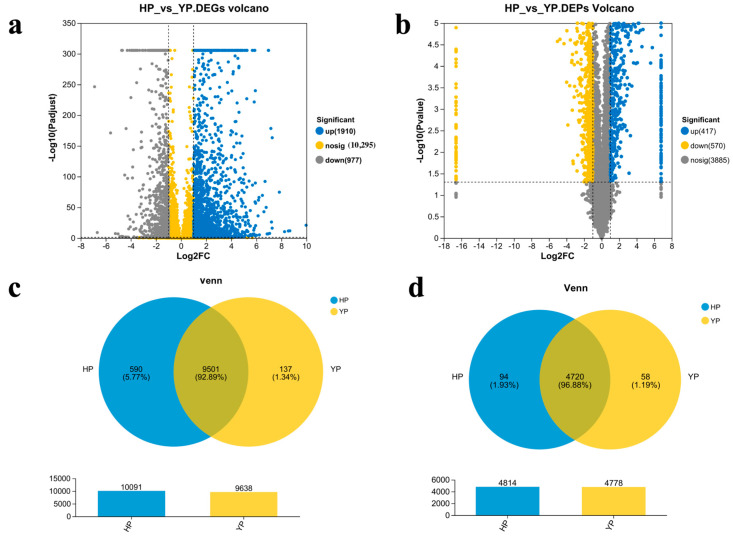
Volcano diagrams: (**a**) DEGs; (**b**) Venn diagram (**c**) DEGs; (**d**) DEPs.

To further assess the molecular mechanisms of *M. sextelata* in YP vs. HP, results regarding the DEGs and DEPs of different comparison groups are displayed in scatter diagrams, which intuitively show the overall distributions of genes and proteins with significantly differential expressions among different comparison groups. In Figure 2, the x-axis shows the fold changes in gene expressions in different samples (log_2_(FC)), while the y-axis shows the significance level of gene expression differences (−log_10_(*q*-value)). It can be seen in Figure 2a that there are 2887 DEGs in YP vs. HP, including 1910 up-regulated genes and 977 down-regulated genes. In Figure 2b, there are 987 DEGs in YP vs. HP, including 417 up-regulated proteins and 570 down-regulated proteins. In Figure 2c, we see that the DEGs in YP vs. HP feature 9501 common differential genes. In Figure 2d, the DEPs in YP vs. HP feature 4720 common differential genes.

### 3.3. GO Functional Enrichment and KEGG Metabolic Pathway Enrichment Analysis

#### 3.3.1. GO Functional Enrichment

**Figure 3:**
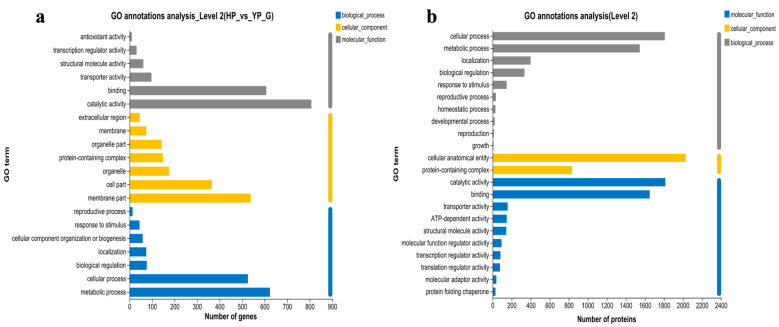
Top 20 GO annotations: (**a**) DEGs and (**b**) DEPs.

In this study, the GO functional annotation classification of DEGs and DEPs in fruiting bodies at YP vs. HP was carried out, and a statistical analysis of the results was conducted. They were divided into three classes—biological process (BP), molecular function (MF), and cellular component (CC)—describing the BP that the gene code products participate in, the MF possessed, and the cell environment involved, respectively. A statistical analysis of the gene numbers in each significantly enriched GO term was implemented, and secondary classification was performed. The first 20 terms of each class are displayed in histograms. It can be seen from Figure 3 that BP shows common significant enrichment in metabolic processes and cellular processes in YP vs. HP. For MF, key functions like catalytic activity and binding are significant. Although there are some differences in CC, the DEGs are mainly located in the membrane and cell parts, while DEPs are mainly correlated with cellular anatomical entities and the protein-containing complex. These results generally disclose important characteristics and trends regarding the DEGs and DEPs related to functions in YP vs. HP, and offer strong support for further explorations of the biological significance and functional difference.

#### 3.3.2. KEGG Functional Enrichment

**Figure 4:**
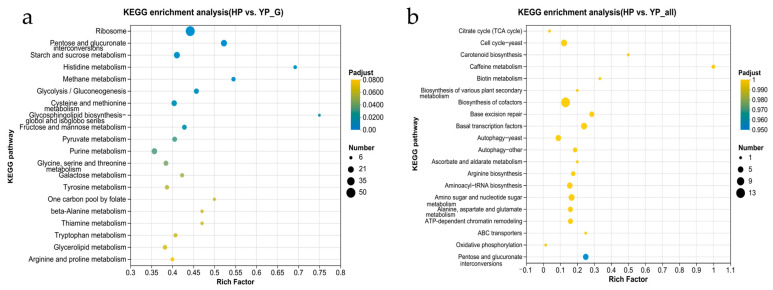
Top 20 significantly enriched KEGG pathways: (**a**) DEGs and (**b**) DEPs.

The first 20 significantly enriched KEGG pathways were carefully classified and compared according to KEGG pathway types (Figure 4). This comparative analysis discloses the significant differences in DEGs and DEPs between YP and HP regarding metabolic pathways. In the KEGG analysis of DEGs shown in Figure 4a, the main significant enrichments can be seen in ribosome, starch and sucrose metabolism, and purine metabolism. The enrichment of these pathways might suggest significant changes in the synthesis and function of ribosome, starch and sucrose metabolism, purine metabolism, and other biological processes during the growth of *M. sextelata* from YP to HP. Such changes have an important influence on the growth and development of *M. sextelata.* In the KEGG analysis of DEPs shown in Figure 4b, we mainly see significant enrichments in the biosynthesis of cofactors, cell cycle of yeast, and autophagy of yeast. Enrichments of these pathways might disclose significant changes in the synthesis and use of cofactors, cell cycle regulation, autophagy processes, and other key biological processes during the growth of *M. sextelata.* These changes might affect the functions and expression modes of proteins.

#### 3.3.3. GO Function and KEGG Functional Enrichment Analysis

**Figure 5:**
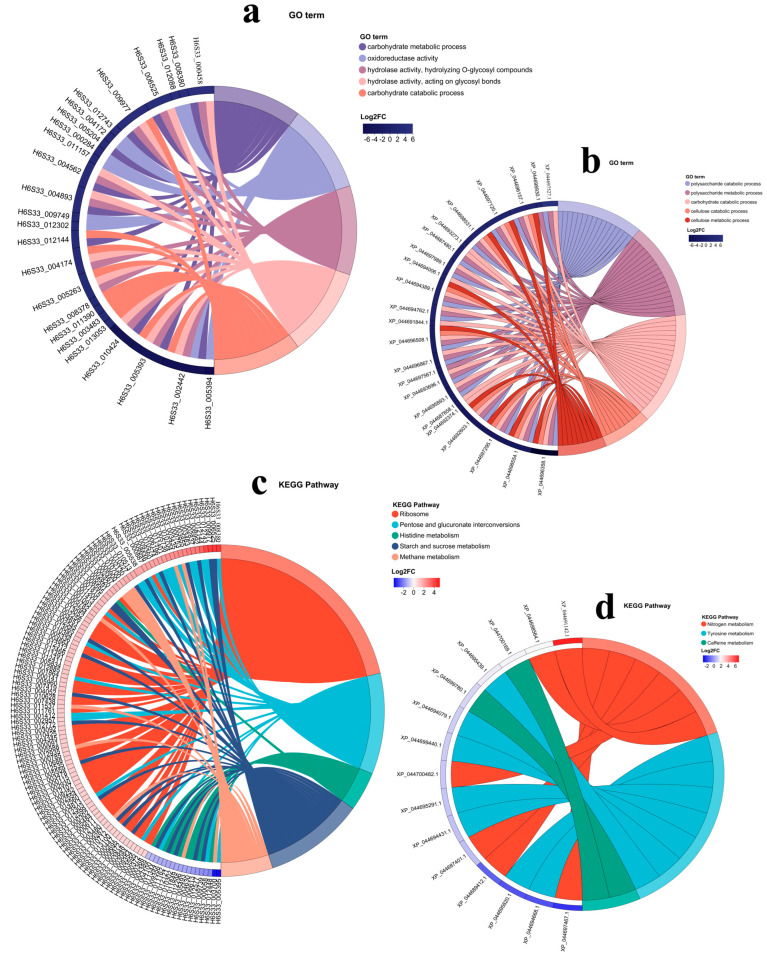
Top five most significantly enriched GO pathways in the enrichment chordal graph: (**a**) DEGs and (**b**) DEPs. Top five most significantly enriched KEGG pathways: (**c**) DEGs and (**d**) DEPs.

In seeking a better understanding of key genes, as well as their biological functions and regulatory network, in the YP and HP of *M. sextelata*, the genes that might play important roles in growth as well as their expression modes in different development phases can be identified more clearly using an enrichment chordal graph. In Figure 5, the sectors in different colors represent different biological processes. The sizes and color intensities of lines reflect the enrichment degree and expression changes in genes in a specific pathway. In Figure 5a, the top five GO terms enriched in the DEGs of YP vs. HP are the carbohydrate metabolic process, oxidoreductase activity, hydrolase activity, hydrolyzing O-glycosyl compoundshydrolase activity, acting on glycosyl bonds, and carbohydrate catabolic process, among others. In Figure 5b, the top five GO terms enriched in the DEPs of YP vs. HP are the polysaccharide catabolic process, polysaccharide metabolic process, carbohydrate catabolic process, cellulose catabolic process, and cellulose metabolic process, among others. In Figure 5c, the top five KEGG pathways enriched in the DEGs of YP vs. HP are ribosome, pentose and glucuronate interconversions, histidine metabolism, starch and sucrose metabolism, methane metabolism, and other pathways. The enrichment of these pathways reflects the diversity of energy metabolism, amino acid metabolism, and carbon metabolism during the growth of *M. sextelata.* In Figure 5d, the top five KEGG pathways enriched in the DEPs of YP vs. HP are nitrogen metabolism, tyrosine metabolism, caffeine metabolism, and others. The enrichment of these pathways might disclose the use and metabolism characteristics of specific nutrients during the growth of *M. sextelata.*

### 3.4. RT-qPCR Verification

**Figure 6:**
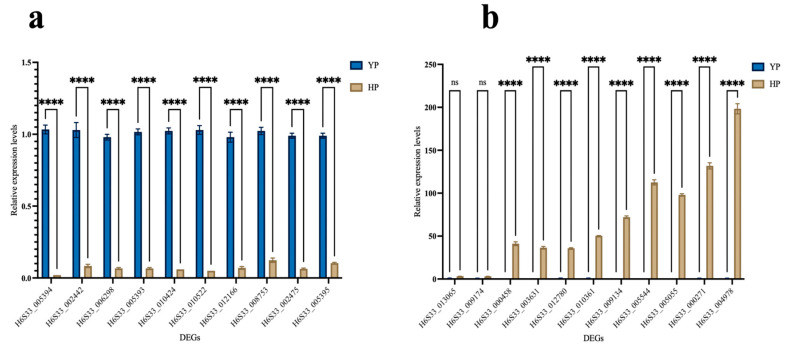
RT-qPCR verification: (**a**) down-regulated DEGs; (**b**) up-regulated DEGs (the testing method was two-way ANOVA and Tukey; ns means *p* > 0.05, indicating insignificant differences; **** means *p* ≤ 0.0001, indicating significant differences).

The RT-qPCR test results in Figure 6 are consistent with the key DEGs shown in the RNA-seq analysis in Supplementary Table S2, indicating that the RNA-seq results are reliable. The expression levels of the genes shown in Figure 6a were all significantly higher under theYP than under the HP. The expression levels of the genes shown in Figure 6b were all significantly higher at the HP than at theYP. The expression levels of these genes under the YP may be very low, resulting in them not being obvious or even visible in the Figure 6b. This may be due to the strong repressive effect of these genes under the YP, or the expression of these genes under the YP itself is very low. Despite the low expression levels under the YP, the up-regulation of these genes under HP was significant, suggesting that these genes may have important biological functions under the HP.

The TPM of H6S33_013065 in HP is about 3.04 times that in YP (290.25/95.35). Such significantly up-regulated expression demonstrates that the starch and saccharose metabolism pathways become more active from the YP to the HP of *M. sextelata*, and they provide more energy and carbon sources to promote the maturity and development of fruiting bodies [32]. Therefore, saccharose plays an important role in the normal development and stress resistance of fruit glucose metabolism [33]. The TPM of H6S33_010424 in HP is about 0.05 times that in YP (2.56/48.18). In other words, the TPM in YP is about 20 times that in HP, showing significantly down-regulated expressions. This implies that the fructose and mannose metabolism pathways might not be the major energy sources in the late growth phase of *M. sextelata*, or that these metabolism pathways are replaced by other more active metabolism pathways.

H6S33_009134, H6S33_005544, H6S33_005055, and H6S33_004978 are annotated as overall components in GO [34]. Their average TPM in YP is extremely low (0.36), but it increases sharply to 48.7 in HP, a difference of about 135 times. Such changes might imply that these genes play a crucial role in the structural and functional adjustment of the cell membrane from the YP to the HP of *M. sextelata.*

### 3.5. Protein–Protein Interaction (PPI) Analysis

**Figure 7:**
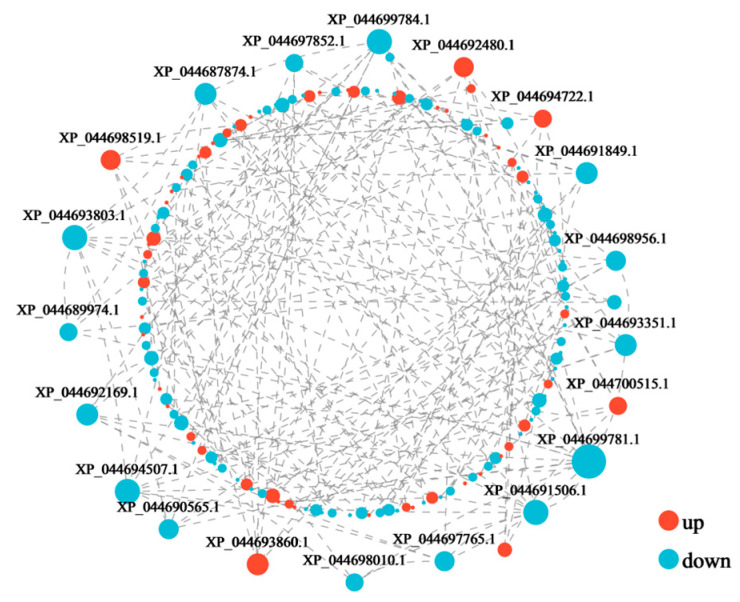
PPI network of top 200 DEPs (The size of the circle represents the level of connectivity; the larger the circle, the higher the connectivity.)

The current study found that the first 200 proteins were dominated by down-regulated genes (Figure 7). Of the top 20 protein pathway definitions, 10 were spliceosome, and 6 were not recognized (Table 1). The spliceosome is a complex ribonucleoprotein complex consisting of five small nuclear ribonucleic acids (snRNAs) and many protein factors. These components work in concert to ensure a correct splicing process. Specifically, the spliceosome excises introns from precursor mRNAs and joins exons in a series of steps, ultimately generating mature mRNAs [35]. This process is essential for the production of mature mRNA molecules since only spliced mRNAs can be translated into proteins. In morel mushrooms, the function of the spliceosome may be related to its stage of growth and development, affecting its morphological construction and metabolic activities. Although there is no direct information available on the specific working mechanism of the RNA spliceosome in *M. sextelata*, it can be inferred that the spliceosome plays a central role in the regulation of gene expression in *M. sextelata*, ensuring that the cell produces the correct proteins according to environmental conditions and the developmental stage.

## 4. Discussion

### 4.1. Analysis of Pathway Metabolic Diversity

During the growth and development of *M. sextelata*, metabolic pathways such as carbohydrate metabolism, polysaccharide and cellulose metabolism, glycometabolism, amino acid metabolism, and nitrogen metabolism play crucial roles. The integration of these metabolic pathways provides information important to understanding the physiological needs and adaptation mechanisms of morel mushrooms at different developmental stages. By delving into these metabolic pathways, we can reveal how morel mushrooms regulate their growth and development to adapt to different environmental conditions. These findings help us understand how morel mushrooms optimize growth and development by regulating their metabolic pathways and how they adapt to environmental changes through a diversity of metabolic pathways.

#### 4.1.1. Glycometabolism

So far, the accepted glycometabolism pathways include sucrose metabolism, sorbitol metabolism, hexose metabolism, and starch metabolism [36]. In the transcriptome study of *M. sextelata*, two genes, H6S33_013065 and H6S33_010424, were annotated to be involved in the starch and sucrose metabolic pathways and the fructose and mannose metabolic pathways, respectively. They showed distinct TPM trends in YP and HP, which implies their important roles in the growth and development of *M. sextelata* and their adaptive changes in different environments.

H6S33_013065 is involved in starch and sucrose metabolism, which may be critical for energy storage and utilization in morel mushrooms. Starch, as the main energy storage substance in plants [37], may provide necessary energy support during the growth and development of *M. sextelata*. Sucrose, on the other hand, is not only a source of energy [38] but may also be involved in physiological processes such as signaling [39]. H6S33_010424, on the other hand, is related to the metabolism of fructose and mannose, which are monosaccharides that play important roles in cell wall synthesis [40], and osmotic pressure regulation, among others processes. Therefore, changes in the expressions of these two genes may directly affect the growth rate, morphogenesis, and stress tolerance of *M. sextelata*.

Although both H6S33_013065 and H6S33_010424 are involved in the sugar metabolism process of *M. sextelata*, there are obvious differences between them. This difference may stem from their different positions and functions in the metabolic network, as well as their response mechanisms to different environmental conditions. Future studies can further explore the specific mechanisms of the roles of these two genes in the growth and development of *M. sextelata* and how they interact with other metabolic pathways to jointly regulate the physiological functions and growth status of *M. sextelata*.

#### 4.1.2. Selenocompound Metabolism

H6S33_002475 is a multifunctional gene involved in several metabolic pathways, including selenium compound metabolism, monolactam biosynthesis, purine metabolism, and sulfur metabolism. The expression of this gene was significantly down-regulated in YP and HP, which may have a profound effect on metabolite composition, biosynthetic capacity, and energy utilization in *M. sextelata*.

Selenium is an important trace element with anti-oxidative stress and cytoprotective effects [41,42]. H6S33_002475 is involved in the metabolism of selenium compounds, and the down-regulation of its expression may weaken the antioxidant capacity of *M. sextelata* and increase its sensitivity to environmental stresses [43]. This may be related to the adaptive changes undertaken by morel mushrooms in different growth stages or environments.

The biosynthesis of monolactam is closely related to the defense mechanism of *M. sextelata*. The down-regulation of H6S33_002475 expression may imply a reduction in pathogen defense requirements in HP, which may be related to the physiological state and environmental adaptation undertaken during the maturation stage of morel mushrooms. This change may reflect the adjustment of pathogen defense strategies in *M. sextelata* at different growth stages. Purine metabolism is critical for nucleic acid synthesis and cell division. The down-regulation of H6S33_002475 expression may affect the rate of nucleic acid synthesis and cell division in *M. sextelata*, which, in turn, affects its growth and development. This effect may be related to the changes in growth rate and morphologic development of *M. sextelata* under different growth conditions. Sulfur is an important component of proteins and enzymes and plays a key role in cell growth and development. H6S33_002475 is involved in sulfur metabolism, and the down-regulation of its expression may disturb the protein synthesis and metabolic activities of *M. sextelata*, which, in turn, may affect its overall physiological functions and growth status. This change may be related to the changes in protein and enzyme requirements of *M. sextelata* under different growth conditions.

Taken together, the down-regulation of H6S33_002475 expression in *M. sextelata* may have profound effects on multiple metabolic pathways, which, in turn, affect its growth rate, morphological development and substrate maturation. These findings not only reveal the metabolic-adaptive changes undertaken by *M. sextelata* under different growth conditions but also provide important clues for future studies. Future studies can further explore the specific mechanism of action of H6S33_002475 in different growth stages or environments and address how to improve the growth performance and quality of morel mushrooms by regulating the expression of this gene. Meanwhile, the roles of other related genes in the growth and development of morel mushrooms can also be investigated to offer a more comprehensive understanding of their metabolic regulatory networks.

### 4.2. Spliceosome

Splicing bodies are dynamically composed of small nuclear RNAs (snRNAs, U1, U2, U4, U5, U6, etc.) and over 200 protein factors. They are ribonucleoprotein complexes that recognize pre-mRNA splicing sites and catalyze splicing reactions [44]. The spliceosome pathway ensures the efficient processing and maturation of mRNA [45].

In the study of the PPI network in the protein composition of morel mushrooms, 10 out of the top 20 key proteins identified were determined to be related to spliceosome, which is of great significance. These proteins form a tight interaction module in the PPI network, demonstrating their synergistic roles in gene expression regulation and their joint participation in the splicing process of mRNA. In addition, the high connectivity of these proteins indicates that they play a crucial role in maintaining the balance and stability of gene expression in morel mushrooms. This discovery not only provides new clues that elucidate the mysteries of the lives of morel mushrooms but also offers new targets of study in determining their gene expression regulation mechanisms, which may open up new concepts and methods for the genetic improvement and breeding of morel mushrooms. 

Subsequent work will screen out differential proteins for validation. Parallel reaction monitoring (PRM) is a targeted quantitative proteomic research method based on high-resolution and high-precision mass spectrometry targeted quantification technology. It can selectively detect target proteins and peptides and provide accurate absolute or relative quantifications for dozens to hundreds of target proteins. This technology can be used for the validation of widely screened data, such as in obtaining target proteins through quantitative proteomics and validating them through PRM [46].

## 5. Conclusions

*M. sextelata* is a valuable edible mushroom species. Carbohydrate metabolism, glycometabolism, and selenocompound metabolism play critical roles in the growth and development of *M. sextelata.* Transcriptomics and proteomics analyses provide us with tools to derive a deep understanding of the molecular mechanisms of these biochemical processes. However, these genes have undergone no specific functional annotation so far. This is related to insufficient research performed on the growth and development of large ascomycetous fungi. It is speculated that these stage-specific genes might play an important role in the ascocarp morphological formation of ascomycetous fungi. The expressions of specific genes in the vegetative growth stage are relatively low, and only a few have been annotated. The functions of these stage-specific genes still have to be further studied.

H6S33_002475 is annotated as participating in multiple metabolism pathways in KEGG, including selenocompound metabolism, monobactam biosynthesis, purine metabolism, and sulfur metabolism. The average TPM is 230.23 in YP and 16.67 in HP, showing a significant trend of down-regulation by about 13.8 times (230.23/16.67). Specifically, H6S33_002475 participates in selenocompound metabolism, monobactam biosynthesis, purine metabolism, and sulfur metabolism. The down-regulated expression of H6S33_002475 might affect the activity of these metabolic pathways. As a result, the metabolite composition, biosynthesis capacity or energy use of *M. sextelata* might be different in HP and YP. Besides this, the down-regulated expression of H6S33_002475 might affect the growth rate, morphological development or fruiting body maturation of *M. sextelata*, thus influencing its overall yield and quality.

These findings not only provide new clues that elucidate the mysteries of the lives of morel mushrooms, but they also offer new targets for studying their gene expression regulation mechanisms, which may generate new concepts and methods for genetically improving and breeding morel mushrooms. At the same time, more in-depth research into related proteins in the PPI network of morel mushrooms is expected to bring about breakthroughs in biological research into and the application of morel mushrooms.

## Figures and Tables

**Table 1:** Top 20 annotations of DEPs.

Node1_Accession_Id	KO_Name	Pathway_Id	Pathway_Definition
XP_044699784.1	CDC40, PRP17	map03040	Spliceosome
XP_044692480.1	------	------	------
XP_044694722.1	POLE	map03410; map03420; map03030	Base excision repair; Nucleotide excision repair; DNA replication
XP_044691849.1	NOP58	map03008	Ribosome biogenesis in eukaryotes
XP_044698956.1	------	------	------
XP_044693351.1	SNW1, SKIIP, SKIP	map03040	Spliceosome
XP_044700515.1	PLD1_2	map00565; map00564; map04144	Ether lipid metabolism; Glycerophospholipid metabolism; Endocytosis
XP_044699781.1	SF3B1, SAP155	map03040	Spliceosome
XP_044691506.1	SF3A1, SAP114	map03040	Spliceosome
XP_044697765.1	PRPF40, PRP40	map03040	Spliceosome
XP_044698010.1	DDX46, PRP5	map03040	Spliceosome
XP_044693860.1	IMP4	map03008	Ribosome biogenesis in eukaryotes
XP_044690565.1	RBM22, SLT11	map03040	Spliceosome
XP_044694507.1	SYF1, XAB2	map03040	Spliceosome
XP_044692169.1	U2AF2	map03040	Spliceosome
XP_044689974.1	RNF113A, CWC24	------	------
XP_044693803.1	CWC22	------	------
XP_044698519.1	BRX1, BRIX1	------	------
XP_044687874.1	DHX16	map03040	Spliceosome
XP_044697852.1	KRI1	------	------

## Data Availability

The original contributions presented in this study are included in the article and Supplementary Materials. Further inquiries can be directed to the corresponding author.

## Supplementary Materials

The following supporting information can be downloaded at: https://www.mdpi.com/article/10.3390/jof11030192/s1, Table S1: Statistics of RNA-seq data of samples. Table S2: Primer sequences used in fluorescence quantitative PCR; Table S3: TPM values and qPCR values of genes; Supplementary Figure S1: PCA plots of DEGs (a) and DEPs (b).
