# Supplementary figures and images for: A Preliminary Exploration of Transcriptome and Proteomic Changes During the Young and Harvest Periods in Morchella sextelata
Source: J Fungi (Basel). 2025 Mar 2;11(3):192. doi: 10.3390/jof11030192 (PMC11943006; doi:10.3390/jof11030192)

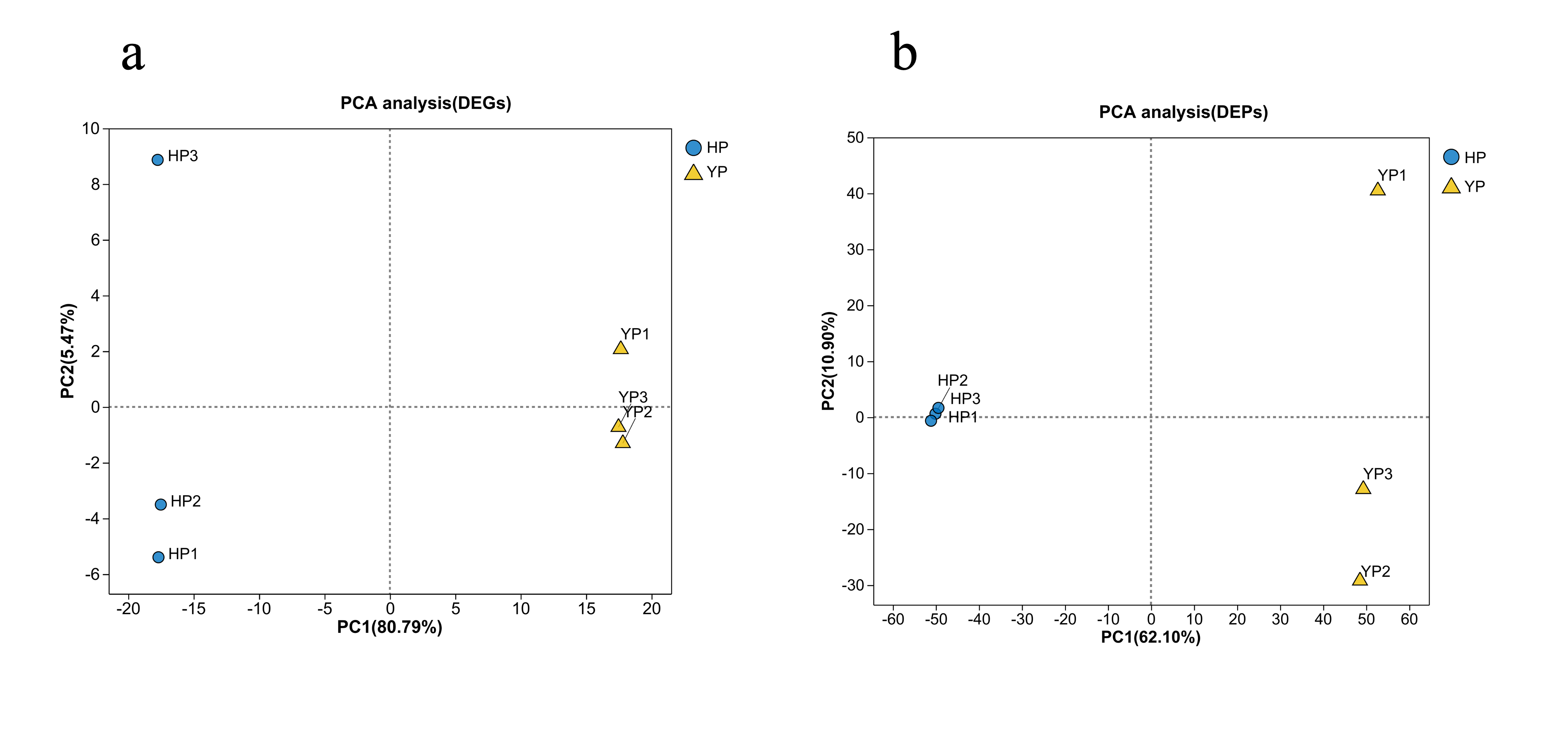

Supplement: Supplementary file 1 [file jof-11-00192-s001.zip › Supplementary Fig.1 .png]
